# Enhanced Optical Switching Characteristics of Organic Phototransistor by Adopting Photo-Responsive Polymer in Hybrid Gate-Insulator Configuration

**DOI:** 10.3390/polym12030527

**Published:** 2020-03-01

**Authors:** Hea-Lim Park, Min-Hoi Kim, Hyeok Kim

**Affiliations:** 1 Department of Materials Science and Engineering, Seoul National University, Kwanak P.O. Box 34, Seoul 151-600, Korea; haelim1017@snu.ac.kr; 2 Department of Creative Convergence Engineering, Hanbat National University, Yuseong-ku, Daejeon 305-719, Korea; mhkim8@hanbat.ac.kr; 3 School of Electrical and Computer Engineering, University of Seoul, 163 Seoulsiripdaero, Dongdaemun-gu, Seoul 02504, Korea

**Keywords:** organic phototransistor, photoresponsive polymer, polymer gate insulator, hybrid gate insulator, optical switching device, poly(4-vinylphenol), poly(methyl methacrylate)

## Abstract

In this study, we developed polymer gate insulator-based organic phototransistors (p-OPTs) with improved optical switching properties by using a hybrid gate insulator configuration. The hybrid gate insulator of our p-OPT has a photoresponsive layer made of poly(4-vinylphenol) (PVP), which enhances the photoresponse, and an interfacial layer of poly(methyl methacrylate) for reliable optical switching of the device. Our hybrid gate insulator-equipped p-OPT exhibits well-defined optical switching characteristics because no specific type of charge is significantly trapped at an interfacial layer/organic semiconductor (OSC) interface. Moreover, our device is more photoresponsive than the conventional p-OPT (here, an OPT with a single-polymer poly(methyl methacrylate) (PMMA) gate insulator), because the characteristic ultraviolet (UV) absorption of the PVP polymer allows the photoresponsive layer and OSC to contribute to the generation of charge carriers when exposed to UV light.

## 1. Introduction

Organic phototransistors (OPTs) are three-terminal organic optoelectronics with an incident light that acts as the external electrode to tune the electrical signal. OPTs not only possess the advantages of organic electronics, such as low cost and mechanical flexibility, but also an advantage of field-effect transistors (i.e., a relatively high signal-to-noise ratio) [1,2,3,4,5,6,7,8,9,10]. To fully exploit the advantages of organic devices (e.g., low cost and mechanical flexibility), it is critical to employ polymer dielectric materials as gate insulators in OPTs. These polymer-based OPTs (p-OPTs) are promising candidates for next-generation portable and wearable optoelectronics.

Among various electromagnetic radiation wavelengths, ultraviolet (UV) light ranging from 10 to 400 nm is harmful to humans, because it is associated with several health problems, such as skin aging, skin cancer, macular degeneration, and cataracts [11,12,13]. Therefore, UV-sensing devices that possess the advantages of a p-OPT configuration (i.e., low cost, flexibility, and a relatively high signal-to-noise ratio), have the potential to become a core technology in the field of healthcare. As photo-sensing devices, the UV radiation-dependent on-off switching function of p-OPTs must be reliable. In addition, for commercialization, the level of performance of p-OPTs should be improved to match that of their inorganic counterparts [14]. In consideration of this, studies purposed to improve the optical switching performance of p-OPTs in a simple and cost-effective way are necessary to facilitate the commercialization of p-OPTs as UV-sensing components in portable and wearable electronic systems.

**Figure 1:**
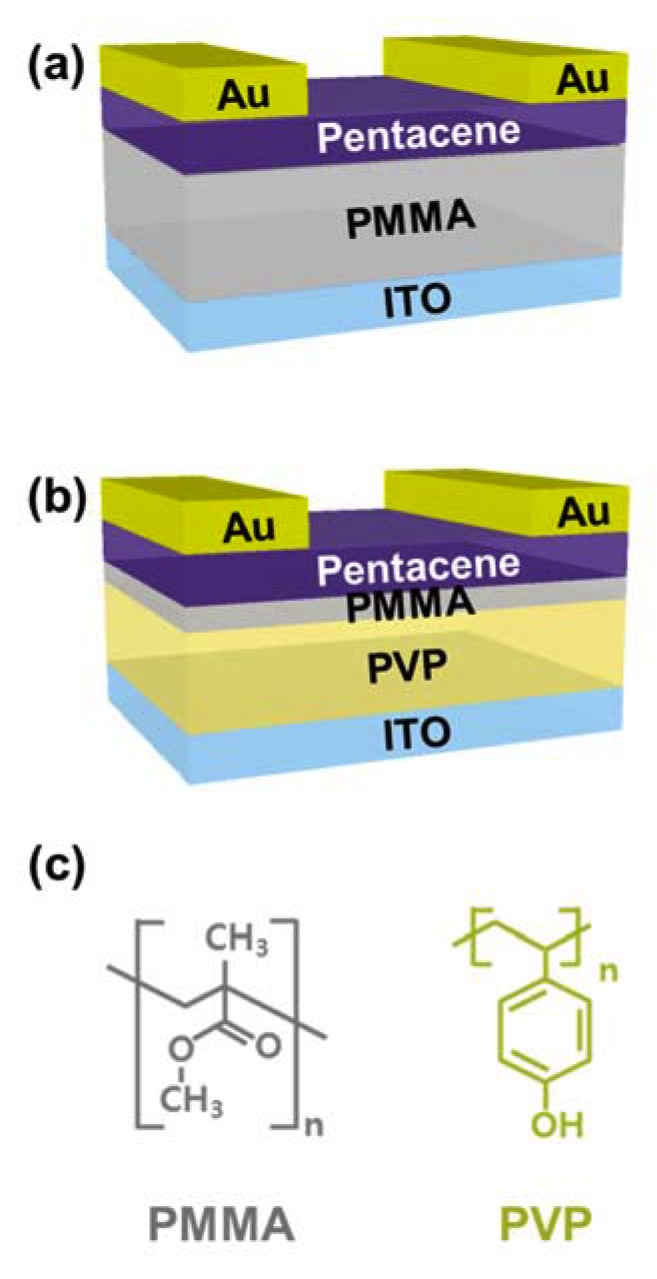
Schematic diagrams of structures of organic phototransistors (OPTs) with (**a**) a single-polymer poly(methyl methacrylate) (PMMA) gate insulator, and (**b**) a hybrid poly(4-vinylphenol) (PVP)/PMMA gate insulator. In the device with the hybrid gate insulator, PVP is a photoresponsive layer, and PMMA is an interfacial layer. (**c**) Chemical structures of PMMA (left) and PVP (right). ITO = indium tin oxide.

In this study, we developed a p-OPT with enhanced optical switching performance by introducing a hybrid gate insulator structure, as shown in Figure 1b. The hybrid gate insulator in our device is composed of two different layers: 1) a photoresponsive layer to enhance photoresponse to UV light, and 2) an interfacial layer for reliable optical switching properties. To construct the photoresponsive layer, the UV-responsive material poly(4-vinylphenol) (PVP) was used. A layer of PVP is suitable as a p-OPT gate insulator because it affords excellent optical and dielectric properties [15,16,17,18,19,20]. However, in such devices, the hydroxyl groups of PVP that act as electron trap sites at the interface between an organic semiconductor (OSC) and a gate insulator inherently limit the optical switching performance [17,18]. We implemented poly(methyl methacrylate) (PMMA) film as the interfacial layer adjacent to the OSC to ensure that the device has reliable optical switching capability, because PMMA has no functional groups for photogenerated minority carriers [17]. Our p-OPT, which has a hybrid gate insulator, exhibited stable UV sensing characteristics, and demonstrated better photosensitivity and photoresponsivity than its conventional counterpart (an OPT with a single-polymer PMMA gate insulator).

## 2. Materials and Methods

To compare the photoresponse of two types of OPTs (i.e., an OPT with a single PMMA layer as a gate insulator, and an OPT with a PVP/PMMA hybrid layer as a gate insulator), two different devices were fabricated as shown in Figure 1a,b. To obtain similar drain levels (electrical characteristics) of both devices, these two types of gate insulators were designed to have different thicknesses, because PMMA and PVP have different dielectric constant (k) values of 3.5 and 4.5, respectively [17]. It should be noted that drain current is related with capacitance per unit area which is proportional to dielectric constant and inversely proportional to thickness of a gate insulator. First, 165-nm-thick indium tin oxide (ITO) layers were patterned on glass substrates and used as gate electrodes. For the OPT with the PMMA-only gate insulator, the PMMA (*M*_w_ = 996,000 g/mol, Sigma-Aldrich, Seoul, Korea) was dissolved in anisole at 7 wt% concentration, and the PMMA solution was spin-coated at 3000 rpm for 60 s to form a 420-nm-thick gate insulator. The PMMA film was then annealed at 110 °C for 2 h under ambient air conditions. To fabricate the hybrid PVP/PMMA gate insulator, PVP solution was prepared by mixing the PVP (*M*_w_ = 25,000 g/mol, Sigma-Aldrich, Seoul, Korea) with poly(melamine-co-formaldehyde) (100 wt% of the PVP) in propylene glycol methyl ether acetate at a concentration of 9 wt%. First, the PVP solution was spin-coated onto the ITO-patterned glass to form 510-nm-thick films that were then annealed at 100 °C for 30 min, and again at 200 °C for 50 min under ambient air conditions. Subsequently, an ultra-thin PMMA film (40 nm) was deposited by spin-coating the PMMA solution (3000 rpm, 60 s) at a concentration of 2 wt%, and then annealed at 110 °C for 2 h under ambient air conditions. On each of these polymer gate insulators, pentacene (50 nm) was thermally evaporated at a rate of 0.5 Å/s under 1 × 10^−5^ Torr of pressure. Au was deposited by inducing thermal evaporation at a rate of 1.0 Å/s under 1 × 10^−5^ Torr of pressure. During the Au deposition process, a shadow mask was used to fabricate source and drain electrodes with a channel length and width of 150 μm and 1 mm, respectively.

The electrical characteristics of the OPTs were measured by operating a semiconductor parameter analyzer (HP4155A, Hewlett–Packard Co., Palo Alto, CA, USA) under ambient air conditions. A UV light source (GL-155, UVSMT, Gyeonggi-do, Korea) with a peak wavelength of 365 nm and intensity of 3 mW/cm^2^ was used to measure the photoresponse of the devices. 

## 3. Results

**Figure 2:**
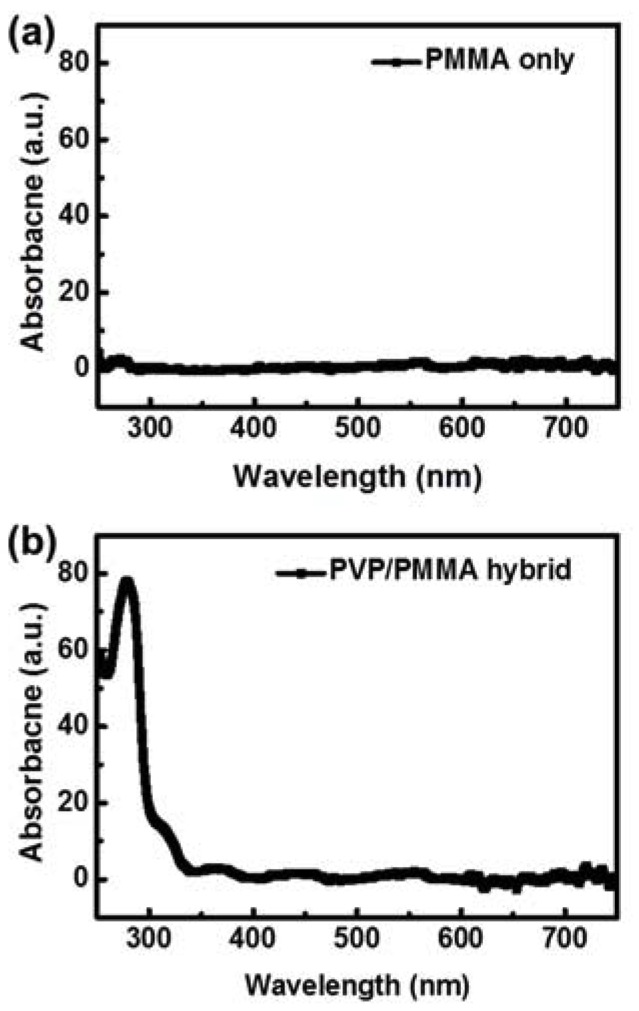
UV–Vis absorbance curves for the (**a**) PMMA layer and (**b**) PVP/PMMA hybrid layer.

To investigate the effects of the hybrid gate insulator configuration on the UV-sensing properties of the p-OPTs, we fabricated two types of devices with different polymer gate insulators (i.e., one type has a single-polymer gate insulator consisting of PMMA, whereas the other type has a hybrid gate insulator consisting of PVP/PMMA as shown in Figure 1a,b, respectively). Since the OSC/gate insulator interface plays a critical role in determining the photoresponsive characteristics of p-OPTs (i.e., the optical switching and memory properties), there have been numerous studies on how to regulate the characteristics of the device by modifying the interface between the OSC and gate insulator [17,18,21,22,23,24,25,26,27]. Specifically, PMMA-based p-OPTs have been reported as promising candidates for photo-switching devices because there are no trapping sites at the OSC/gate insulator interface, as shown in the left panel of Figure 1c [17]. Therefore, we chose to use the p-OPT with a single-polymer PMMA gate insulator as a reference device for evaluating the optical switching performance of the device with the proposed hybrid gate insulator. The reasons for implementing PVP as a photoresponsive layer in the hybrid gate insulator configuration are as follows. First, PVP has been widely explored as a gate insulating material in the field of organic electronics owing to its excellent insulating capability [15,16]. Secondly, PVP has the unique ability to absorb UV light [18,19,20]. This characteristic enhances the photoresponse of p-OPTs. On top of the PVP gate insulator, PMMA was deposited adjacent to the OSC as an interfacial layer to realize photo-switching characteristics [17]. Note that the p-OPTs with the gate insulator consisting of PVP exhibited optical memory behavior because of the electron trapping sites created by the PVP hydroxyl groups, as shown in the right panel of Figure 1c [17,18,19,24]. Figure 2 shows the UV–Vis absorbance of the PMMA layer and PVP/PMMA hybrid layer. The PMMA barely absorbed the light in the range of 250 to 750 nm, whereas absorption of the hybrid layer began near 340 nm before sharply increasing and reaching its peak at the wavelength of 280 nm. Thus, the PVP layer enabled UV light absorption of the PVP/PMMA hybrid layer.

**Figure 3:**
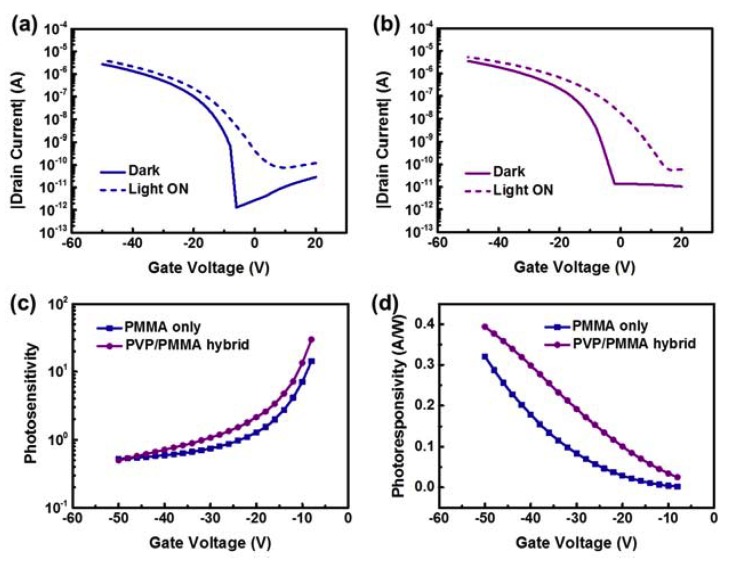
Transfer curves for the OPT with the (**a**) single-polymer PMMA gate insulator, and (**b**) hybrid PVP/PMMA gate insulator in the UV-exposed and non-exposed states. (**c**) Photosensitivity and (**d**) photoresponsivity of the devices as a function of gate voltage.

We examined the static photoresponsive behavior of the devices. As shown in Figure 3a,b, the transfer curves for the two types of p-OPTs in the UV-exposed and non-exposed states were obtained under the conditions of a gate voltage (*V*_g_) range of 20 to −50 V and drain voltage (*V*_d_) of −50 V. Note that, as for off current in the dark, the hybrid type exhibited a somewhat higher value than the single PMMA device. This may be attributed to the hydroxyl groups of the PVP in the hybrid type which responded to moisture in the air during measurement [28,29]. The onset voltage shift (Δ*V*_on_) of a device exposed to light has previously been used to analyze photoresponse [17,18,19,22]. The Δ*V*_on_ values of the devices with the single-polymer gate insulator and hybrid gate insulator were 14 and 18 V, respectively. This means that, in terms of Δ*V*_on_, the photoresponse of the device with the hybrid gate insulator was 1.29 times higher than that of the reference device with the single-polymer insulator. Figure 3c,d respectively shows the photosensitivity and photoresponsivity of the devices in the on state. In terms of photosensitivity and photoresponsivity, the hybrid gate insulator-based device demonstrated better performance than the reference device. 

**Figure 4:**
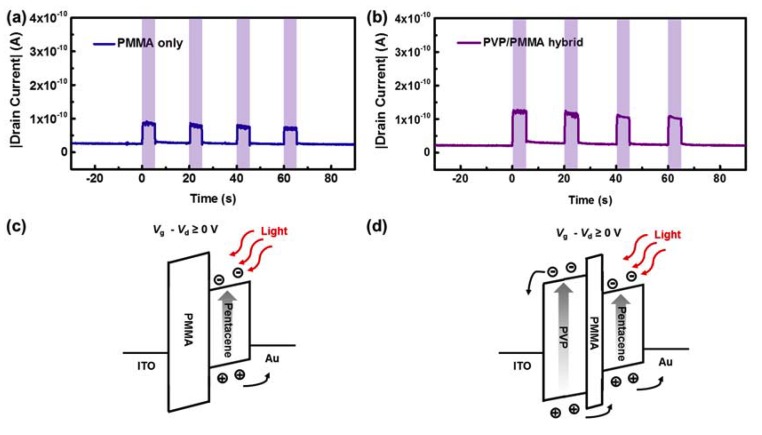
Dynamic photoresponsive behavior of the OPTs with the (**a**) single-polymer PMMA gate insulator and (**b**) hybrid PVP/PMMA gate insulator. The shaded areas indicate the state of the device when exposed to UV light. Schematic diagrams illustrating the operating principles of the OPTs with the (**c**) single-polymer PMMA gate insulator and (**d**) hybrid PVP/PMMA gate insulator.

The real-time photoresponse of the p-OPTs can be examined. The dynamic photoresponsive behavior of each device was determined by evaluating the drain current as a function of time under the operating conditions of a 5 s duration of UV light exposure applied in 15 s intervals, as shown in Figure 4a,b. The shaded areas indicate periods of UV light exposure. Both p-OPTs exhibited well-defined UV radiation-dependent optical switching behavior. Specifically, the drain current abruptly increased immediately after UV radiation was applied; this phenomenon is mainly attributable to the photogenerated electron hole pairs. During periods of UV exposure (i.e., the shaded areas in Figure 4a,b), both types of devices maintained relatively constant current levels. This implies that the number of both electrons and holes trapped at the PMMA/OSC interface of each UV light-exposed device was not exceptionally large in these devices, even though the PMMA-based organic field-effect transistors were reported to have hole trapping properties [30,31]. After the UV light was turned off, the drain current immediately returned to its initial state. During periods of UV exposure, the constant current levels of the devices with the single-polymer insulator and hybrid gate insulator were 7.98 × 10^−11^ and 1.17 × 10^−10^ A, respectively. Thus, the photoresponse of the device with the hybrid gate insulator was 1.47 times higher than that of the reference device. To analyze the operating principles of the devices, an energy band diagram was constructed for each device; they are schematically illustrated in Figure 4c,d [27]. As shown in the diagram for the reference device with a single-polymer PMMA insulator (Figure 4c), electron-hole pairs were only generated in the pentacene layer when the device was exposed to UV light. Under the condition of an applied electric field, as created by the source and drain electrodes, the photogenerated charges were separated into electrons and holes, which were then extracted to the source and drain electrodes, respectively. In the case of the hybrid gate insulator (Figure 4d), electron-hole pairs were generated in the pentacene and PVP layers when the device was exposed to UV light. The charge behaviors in the pentacene layers of both devices were the same. However, in the UV-responsive PVP layer, photogenerated holes were tunneled through the ultra-thin PMMA layer, and extracted to the drain electrode, contributing to higher photocurrent; alternatively, the photogenerated electrons migrated to the gate electrode [32,33]. Thus, the use of the UV-responsive PVP layer and interfacial layer of PMMA, which does not have trapping sites, allows our proposed hybrid gate insulator configuration to yield p-OPTs with stable optical switching characteristics and better photoresponse than the conventional optical switching device (the OPTs with the single-polymer PMMA gate insulator). Note that the conventional optical switching device would further be expanded to the previously reported OPTs which had gate insulators of not only PMMA, but also polystyrene, amorphous fluoropolymer CYTOP, poly (4-phenoxymethyl styrene), and various types of self-assembly monolayers [17,23,24,34,35].

## 4. Conclusions

We presented a simple and effective approach for improving the UV-sensing properties of p-OPTs through the introduction of a hybrid gate insulator structure. To fabricate the hybrid gate insulator, the PMMA, which has well-defined optical switching characteristics without showing a specific type of charge carriers, was formed on top of the UV-responsive PVP layer. It was found that, in the device with the hybrid gate insulator, the UV-responsive PVP layer and OSC contribute to the generation of charge carriers when the p-OPT is exposed to UV light; this is because of the unique characteristic of UV absorption of the PVP polymer. In contrast, in the conventional device of the optical switching characteristics (i.e., the single-polymer PMMA device), electron-hole pairs were only photoinduced in the OSC. The device with the hybrid gate insulator exhibited well-defined optical switching behavior in response to the on/off switching of the UV light. Moreover, it demonstrated better photoresponse than the conventional device. Our simple approach for enhancing the photoresponse of p-OPTs by adopting the proposed hybrid gate insulator configuration can provide a useful guideline for the design and construction of high-performance, organic-based photo-switching components for next-generation portable and wearable optoelectronics.
